# Lymph node dissection in lung cancer surgery: a comparison between robot-assisted vs. video-assisted thoracoscopic approach

**DOI:** 10.3389/fsurg.2024.1395884

**Published:** 2024-06-17

**Authors:** Patrick Deniz Hurley, Giulia Fabbri, Nabih Berjaoui, Akshay Jatin Patel, Savvas Lampridis, Tom Routledge, Andrea Bille

**Affiliations:** ^1^ Department of Thoracic Surgery, Royal Papworth Hospital NHS Foundation Trust, Cambridge, United Kingdom; ^2^ Department of Thoracic Surgery, Guy’s and St Thomas’ NHS Foundation Trust, London, United Kingdom; ^3^ Institute of Immunology and Immunotherapy, University of Birmingham, Birmingham, United Kingdom

**Keywords:** lymph node dissection, lung cancer, robot-assisted surgery, video-assisted surgery, thoracotomy

## Abstract

**Background:**

TNM staging is the most important prognosticator for non-small cell lung cancer (*N*SCLC) patients. Staging has significant implications for the treatment modality for these patients. Lymph node dissection in robot-assisted thoracoscopic (RATS) surgery remains an area of ongoing evaluation. In this study, we aim to compare lymph node dissection in RATS and VATS approach for lung resection in NSCLC patients.

**Methods:**

We retrospectively compiled a database of 717 patients from July 31, 2015–July 7, 2022, who underwent either a wedge resection, segmentectomy or lobectomy. We analysed the database according to lymph node dissection. The database was divided into RATS (*n* = 375) and VATS (*n* = 342) procedures.

**Results:**

The mean number of lymph nodes harvested overall with RATS was 6.1 ± 1.5 nodes; with VATS approach, it was 5.53 ± 1.8 nodes. The mean number of N1 stations harvested was 2.66 ± 0.8 with RATS, 2.36 ± 0.9 with VATS. RATS approach showed statistically higher lymph node dissection rates compared to VATS (*p* = 0.002). Out of the 375 RATS procedures, 26 (6.4%) patients undergoing a RATS procedure were upstaged from N0/N1 staging to N2. N0/N1–N2 upstaging was reported in 28 of 342 (8.2%) patients undergoing a VATS procedure. The majority of upstaging was seen in N0–N2 disease: 19 of 375 (5%) for RATS and 23 of 342 (6.7%) for VATS.

**Conclusions:**

We conclude that in RATS procedures, there is a higher rate of lymph node dissection compared to VATS procedures. Upstaging was mostly seen in N0–N2 disease, this was observed at a higher rate with VATS procedures.

## Introduction

The eighth edition of the TNM classification for staging lung cancer was derived from a retrospective analysis of 94,708 patients with diagnosed lung cancer (1). A study conducted by Asamura et al. that analysed 26,326 patients with any T staging category and no metastasis (M0), showed a difference in prognosis in neighbouring pathological N (pN) categories. The 5-year survival rate according to pN status was 75% for N0, 49% for N1, 36% for N2, and 20% for N3 (2). The N stage is the most significant prognosticator in lung cancer surgery. A robust lymph node evaluation is therefore imperative to optimise oncological outcomes for curative intent lung surgery.

The International Association for the Study of Lung Cancer (IASLC) guidelines consider adequate dissection of lymph nodes as stations 2R, 4R, 7, 10R, and 11R for right-sided tumours and 5, 6, 7, 10L, and 11L for left-sided tumour. Additionally, they recommend that station 9 be dissected in patients undergoing a lung cancer resection involving the lower lobe.

The efficacy of lymph node dissection with a VATS approach has been shown to have equivalent outcomes with an open thoracotomy approach (3, 4). A meta-analysis by Zhang and colleagues comparing VATS and open lung resections for stage 1 lung cancer found fewer total lymph nodes were dissected in the VATS group compared to the open group (5). Comparisons between VATS and RATS have demonstrated non-inferior lymph node dissection in RATS (6, 7). Toker and colleagues having compared open, VATS and RATS reported greater N1 dissection with RATS approach (8). Robot-assisted thoracic surgery has gained wider adoption in this timeframe and the evaluation of optimal lymph node dissection remains (6–8); hence we aim to add to the literature by comparing lymph node dissection in RATS, and VATS lung resection for NSCLC patients.

## Methods

### Patient selection

We compiled a retrospective database on patients with NSCLC who underwent anatomical lung resections. The database is composed of 717 patients who underwent a lung resection and lymph node dissection via video-assisted or robot-assisted approach between July 2015 and July 2022 at a single tertiary centre, Guy's Hospital, London. All patients underwent a preoperative evaluation to assess fitness for surgery. An endobronchial ultrasound (EBUS) was performed when the lesion was amenable to endoscopic biopsy, and this was employed for concurrent invasive mediastinal staging. In cases of inconclusive results with EBUS, formal surgical mediastinoscopy (not VAMLA) was employed particularly in higher (III) stage disease or high PET mediastinal avidity. All patients underwent a Computer Tomography (CT) and Fluoro-deoxyglucose F18 (FDG) Positron Emission Tomography (PET) imaging to assess the uptake and location of the tumour. The surgical approach was decided by the operating surgeon and discussed with the patient. A minimally invasive approach was selected if technically feasible.

### Surgical technique

All lung resections were performed by two board-certified thoracic surgeons and have achieved the appropriate competence to independently conduct robotic operations. There is a dedicated team for robotic surgery including surgical nurse practitioners that aid in the bedside role of robotic surgery. RATS resections were performed using a Da Vinci Xi Surgical Robot (Intuitive Surgical, Inc., Santa Clara, CA, USA) via 4 robotic ports (two 8 mm ports and two 12 mm ports) plus an additional port for bedside assistance and specimen retrieval. VATS lobectomies were performed with a three-port approach. Each operation included a systematic lymph node dissection in adherence to IASLC and European Society of Thoracic Surgeons guidelines. The dissection included stations 2R, 4R, 7, 10R, and 11R for right-sided tumours and 5, 6, 7, 10L, and 11L for left-sided tumours was complete when amenable. Station 9 was dissected in patients with lower lobe tumours. Our unit-specific definition of lymph node dissection was resection of all visualised lymph node tissue within a particular anatomical site (IASLC classification) that was considered to be safe and accurate.

## Data collection

### Inclusion criteria

We included patients who underwent a wedge resection, segmentectomy, or lobectomy.

### Exclusion criteria

Pneumonectomies as well as open operations were excluded.

Patient demographics, operative reports and pathology reports were obtained for each patient. The lymph nodes that were dissected were categorised according to lymph node stations classified by the IASLC in the database. The database was subsequently divided into a RATS group (*n* = 375), a VATS group (*n* = 342). All two groups were subdivided based on the laterality of the operation.

## Statistical analysis

We analysed the database using Excel software (Microsoft Corp, Seattle, WA). Between-group differences were evaluated using the Chi-test for categorical variables and the Wilcoxon-Mann-Whitney test for continuous variables. All statistical tests were two-tailed, and *p* values < 0.05 were considered statistically significant. All analyses were carried out using GraphPad Prism version 9.5.1 (528).

## Results

The total number of operations in the database was 717. The RATS group accounted for 375 (52.3%) operations (223 right-sided and 152 left-sided), the VATS group accounted for 342 (47.7%) operations (205 right-sided and 137 left-sided).

Out of the 717 operations, 570 (79.5%) of the operations were lobectomies. This was followed by 117 (16.3%) segmentectomies and 30 (4.1%) wedge resections. The RATS group accounted for 313 of the lobectomies, this was measured 257 times via VATS approach. There were 58 RATS segmentectomies compared to 59 VATS segmentectomies.

### Comparison of total lymph node dissection

Table 1 shows the overall mean number of lymph nodes harvested in the two subgroups. The mean number of lymph nodes harvested in the RATS approach was 6,03 ± 1,5. For right-sided procedures by RATS approach the mean number of LNs dissected was 6,10 ± 1,5. Left-sided procedures yielded a mean of 5,93 ± 1,4.

**Table 1:** Total number of lymph nodes dissected.

	VATS	RATS
Overall	5,52 ± 1,7	6,03 ± 1,5
N1 Stations	2,36 ± 0,9	2,66 ± 0,8
N2 Stations	3,17 ± 1,5	3,41 ± 1,2
Right sided operations		
Overall	5,53 ± 1,8	6,10 ± 1,5
N1 Stations	2,30 ± 0,9	2,55 ± 0,7
N2 Stations	3,23 ± 1,3	3,57 ± 1,2
Left sided operations		
Overall	5,51 ± 1,6	5,93 ± 1,4
N1 Stations	2,44 ± 0,9	2,74 ± 0,7
N2 Stations	3,09 ± 1,0	3,18 ± 1,1

Values are given in mean ± standard deviation.

For VATS approach, the mean number of lymph node dissection was found to be 5,52 ± 1,7. For right-sided procedures, this was 5,53 ± 1,8 and for left-sided procedures the mean was 5,51 ± 1,6. These results suggest more lymph nodes were dissected overall in RATS procedures when compared to VATS approach (*p* = 0.002).

### Comparison of N1 lymph node dissection

The mean number of overall N1 LNs dissected in the RATS group was 2,66 ± 0,8. For VATS approach the number of N1 LNs dissected was 2,36 ± 0,9. The higher level of mean lymph node dissection in RATS showed statistical significance when compared to VATS approach (*p* = 0.001).

Table 2 shows the percentage of operations where an N1 lymph node was dissected for each subgroup. Station 10 lymph nodes were dissected in 89.2% for VATS group and 86.7% for RATS group. Station 11 was dissected most in the RATS group in 90.1% of operations. This was followed by 80.7% of VATS operations.

**Table 2:** N1 lymph node dissection.

	VATS	RATS
Station 10 (*n*,%)	305 (89.2%)	325 (86.7%)
Station 11 (*n*,%)	276 (80.7%)	338 (90.1%)
Station 12 (*n*,%)	229 (67%)	296 (78.9%)

### Comparison of N2 lymph node dissection

The mean number of overall N2 LNs dissected in the RATS group was 3,41 ± 1,2. For the VATS group the overall number of N2 LNs that were dissected was 3,17 ± 1,5. RATS approach yielded an increased mean of LNs when compared to VATS (*p* = 0.041).

Table 3 describes the percentage of lymph node stations dissected in each subgroup. In right-sided operations station 7 was the most dissected lymph node station. This station was dissected 99.1% of operations with RATS approach compared to 88.3% in VATS.

**Table 3:** Mediastinal lymph node dissection.

	VATS	RATS
Right Sided Operations	(*n* = 205)	(*n *= 223)
Superior Mediastinal Nodes (*n*, %)		
Station 2R	142 (69.3%)	143 (64.1%)
Station 4R	177 (86.3%)	180 (80.7%)
Subcarinal Lymph Node (*n*,%)		
Station 7	181 (88.3%)	221 (99.1%)
Inferior Mediastinal Lymph Nodes (*n*,%)		
Station 8R	66 (32.2%)	100 (44.8%)
Station 9R	81 (39.5%)	122 (54.7%)
Left Sided Operations	(*n *= 137)	(*n *= 152)
Paraaortic Lymph Nodes (*n*,%)		
Station 5	128 (93.4%)	119 (78.3%)
Station 6	78 (56.9%)	60 (39.5%)
Subcarinal Lymph Node (*n*,%)		
Station 7	125 (91.2%)	140 (92.1%)
Inferior Mediastinal Lymph Nodes (*n*,%)		
Station 8L	19 (13.9%)	52 (34.2%)
Station 9L	71 (51.8%)	106 (68.7%)

In Tables 4, 5, we illustrate lymph node dissection for lobectomies depending on anatomical location. (Tables 4, 5).

**Table 4:** Right sided lobe specific lymph node dissection.

	VATS	RATS
Right Lower Lobe	(*n *= 53)	(*n *= 56)
Superior Mediastinal Nodes (*n*,%)		
Station 2R	30 (56.6%)	29 (51.7%)
Station 4R	35 (66%)	34 (60.71%)
Subcarinal Lymph Node (*n*,%)		
Station 7	48 (90.5%)	54 (96.42%)
Inferior Mediastinal Lymph Nodes (*n*,%)		
Station 8R	27 (50.9%)	31 (55.35%)
Station 9R	35 (66%)	38 (67.8%)
Right Middle Lobe	(*n* = 25)	(*n *= 10)
Superior Mediastinal Nodes (*n*,%)		
Station 2R	18 (72%)	7 (70%)
Station 4R	22 (88%)	8 (80%)
Subcarinal Lymph Node (*n*,%)		
Station 7	24 (96%)	10 (100%)
Inferior Mediastinal Lymph Nodes (*n*,%)		
Station 8R	5 (20%)	7 (70%)
Station 9R	4 (16%)	6 (60%)
Right Upper Lobe	(*n *= 117)	(*n *= 113)
Superior Mediastinal Nodes (*n*,%)		
Station 2R	88 (75.2%)	80 (70.7%)
Station 4R	113 (96.5%)	108 (95.5%)
Subcarinal Lymph Node (*n*,%)		
Station 7	101 (86.3%)	112 (99.2%)
Inferior Mediastinal Lymph Nodes (*n*,%)		
Station 8R	33 (28.2%)	38 (33.6%)
Station 9R	39 (33.3%)	48 (42.4%)

**Table 5:** Left sided lobe specific lymph node dissection.

	VATS	RATS
Left Lower Lobe	(*n *= 42)	(*n *= 54)
Paraaortic Lymph Nodes (*n*,%)		
Station 5	36 (85.7%)	26 (48.1%)
Station 6	21 (50%)	11 (20.3%)
Subcarinal Lymph Node (*n*,%)		
Station 7	42 (100%)	51 (94.4%)
Inferior Mediastinal Lymph Nodes (*n*,%)		
Station 8L	14 (33.3%)	26 (48.1%)
Station 9L	37 (88%)	46 (85.1%)
Left Upper Lobe	(*n *= 70)	(*n *= 68)
Paraaortic Lymph Nodes (*n*,%)		
Station 5	67 (95.7%)	65 (95.5%)
Station 6	39 (55.7%)	51 (75%)
Subcarinal Lymph Node (*n*,%)		
Station 7	61 (87.1%)	59 (86.7%)
Inferior Mediastinal Lymph Nodes (*n*,%)		
Station 8L	3 (4.2%)	45 (66.1%)
Station 9L	24 (34.2%)	37 (54.4%)

#### Nodal upstaging

Out of the total 717 operations, 670 (93%) were preoperatively staged as T1a–T2b tumours, and the remaining 47 (6.5%) were staged as T3–T4 tumours. The T1a–T2b tumours that were resected by RATS approach totalled to 338 (50.5%), this value was 332 (49.5%) for VATS approach. As for larger T3–T4 tumours, these resection totalled to 37 (78.7%) for RATS and 10 (21.2%) for VATS. Nodal upstaging from N0–N1 was seen in 21 out of 342 (6.1%) of VATS operations and 26 out of 375 (6.9%) RATS operations. In terms of upstaging from N0/N1–N2, VATS resections were upstaged to N2 in 28 of 342 (8.2%) operations. Out of the 28, 23 operations saw an upstaging from N0–N2 and 5 were upstaged from N1–N2. RATS operations were upstaged the least from N0–N2. In 375 operations, 24 operations (6.4%) were upstaged from N0/N1–N2. Out of 24 resections 19 of the operations saw an upstaging from N0–N2 and the remaining 5 were upstaged from N1–N2.

## Discussion

Lymph node dissection remains a topic of investigation in the era of robot-assisted minimally invasive thoracic surgery. The ACOSOG Z0030 trial showed that mediastinal lymph node dissection provides the patient with the most accurate staging and opportunity for adjuvant therapy if occult metastatic disease is present (9).

The N component of the TNM staging is useful for physicians assessing malignancies that have significantly different prognoses. The importance of cancer staging has been compounded with recent trials suggesting the addition of neoadjuvant or adjuvant immunotherapy may improve event-free survival or disease-free survival of lung cancer (10). The FDA has approved the use of nivolumab and atezolizumab as neoadjuvant and adjuvant treatment for stages II and IIIA NSCLC respectively based on the CheckMate 816 and Impower010 trials (11, 12). Staging the patients correctly will enable the identification enabling increasingly effective treatment options.

In our study, we have shown a higher degree of lymph node dissection in RATS operations compared to VATS operations in patients undergoing lung resection for early-stage NSCLC lung cancer. The ROMAN study demonstrated superior lymph node dissection in RATS. The results showed a median number of seven hilar nodes dissected with RATS compared to four with VATS. In addition, there was a median of seven mediastinal nodes dissected by RATS compared to five by VATS. Both findings in the trial were statistically significant (13). The RVLob trial, a randomised control trial comparing robot-assisted lobectomy to video-assisted lobectomy demonstrated a higher degree of lymph node dissection in robot robot-assisted approach (14). Conversely, a meta-analysis by Hu et al. looking at twenty retrospective cohort studies argued that there was no statistically significant advantage between VATS and RATS (15). The risk stratification of the TNM staging relies on proper application, the prognostic value of pN depends on the thoroughness of the assessment (16). In this context, improving surgical nodal staging must be prioritised for optimal oncological outcomes.

A possible explanation for the increased level of dissection in robotic operations is the enhanced range of motion with articulated extensions when dissecting lymph nodes compared to video-assisted operations. Palande et al. reasoned that difficulties with VATS lymph node dissection were mostly due to suboptimal exposure, especially for subcarinal nodes (17). In addition to the broadened range of motion, a three-dimensional view of the operative field in robot-assisted operations equips the operating surgeon with an enhanced visualisation of the lymph node. The different surgical modalities have been compared in the literature. Toker et al. comparing open, VATS and RATS lymph node dissection reported superior N1 lymph node dissection in RATS operations when compared to VATS. The group speculated that the sharp dissection of the vascular sheath around the pulmonary vessels in RATS procedures compared to a blunt dissection in VATS procedures could explain the higher degree of N1 lymph nodes obtained in RATS (8). From a technical perspective, it is feasible to dissect each mediastinal and hilar lymph node station with a VATS approach however a RATS approach provides the surgeon with better imaging and retraction opportunities when compared to VATS.

Nodal upstaging occurs when the patient is found to have more advanced disease compared to the clinical assessment in the preoperative period. A study by Kneuertz et al. comparing 1,053 patients with clinical stage N0/N1 NSCLC undergoing RATS, VATS and open lung resection found that the overall rate of LN upstaging was highest in open thoracotomies (21.8%) followed by RATS (16.2%) and VATS (12.3%) (18). Open procedures have been demonstrated to have a significantly higher rate of upstaging than VATS procedures (19–21). This could be due to the patient selection for open procedures as opposed to minimally invasive procedures. Between VATS and RATS, nodal upstaging from N0 to N1 was seen in 21 out of 342 (6.1%) of VATS operations and 26 out of 375 (6.9%) RATS operations. The difference between the two groups was negligible and not significant. One possible explanation is that a lot of the VATS data is more historic, and performed during a time when mediastinal staging techniques were less developed. Nodal upstaging acts as a surrogate for the extent of lymph node sampling and subsequently effective oncological staging, as such correlation with survival remains a key area of study in the future.

Cost effectivity is a controversial point of discussion, one of the outcomes of the RVLob trial was the higher hospitalisation cost of the robot-assisted approach (14). A detailed micro-cost analysis by Shanahan et al. showed that the increased cost of RATS was primarily driven by the higher cost of consumable equipment and the secondary reason was higher staff expenses (22). The RAVAL trial studying if robot-assisted lobectomy is cost-effective and offers improved patient-reported health utility in early NSCLC when compared to video-assisted lobectomy is ongoing. Early results released in 2023 suggest that robot-assisted lobectomy is cost-effective with similar patient-reported health utility (23).

There are certain limitations to our study these include being a retrospective study with a relatively small sample size of 717 patients. This study was conducted at a single tertiary centre by two surgeons limiting the generalisability of the study and introducing operator dependency to the data presented. The study does not include long-term data on outcomes such as survival or recurrence.

## Conclusion

In this study, we compare lymph node dissection obtained from RATS approach and VATS approach for lung resection of NSCLC. We analysed data on N1/N2 lymph node dissection and nodal upstaging. We conclude that there is a higher rate of overall lymph node dissection in RATS approach when compared to VATS. These findings suggest that RATS may be a more effective approach for achieving adequate lymph node dissection in patients with early NSCLC, however it is important to appreciate that this can be attributed to other factors including the skill of the surgeon and complexity of the procedure, not solely the employed technique.

## Data Availability

The original contributions presented in the study are included in the article/supplementary material, further inquiries can be directed to the corresponding author.
